# Probing biological redox chemistry with large amplitude Fourier transformed ac voltammetry

**DOI:** 10.1039/c7cc03870d

**Published:** 2017-08-14

**Authors:** Hope Adamson, Alan M. Bond, Alison Parkin

**Affiliations:** a Department of Chemistry , University of York , Heslington , York , YO10 5DD , UK . Email: alison.parkin@york.ac.uk; b School of Chemistry , Monash University , Clayton , VIC 3800 , Australia . Email: alan.bond@monash.edu

## Abstract

Biological electron-exchange reactions are fundamental to life on earth. Redox reactions underpin respiration, photosynthesis, molecular biosynthesis, cell signalling and protein folding. Chemical, biomedical and future energy technology developments are also inspired by these natural electron transfer processes. Further developments in techniques and data analysis are required to gain a deeper understanding of the redox biochemistry processes that power Nature. This review outlines the new insights gained from developing Fourier transformed ac voltammetry as a tool for protein film electrochemistry.

## Abstract 2

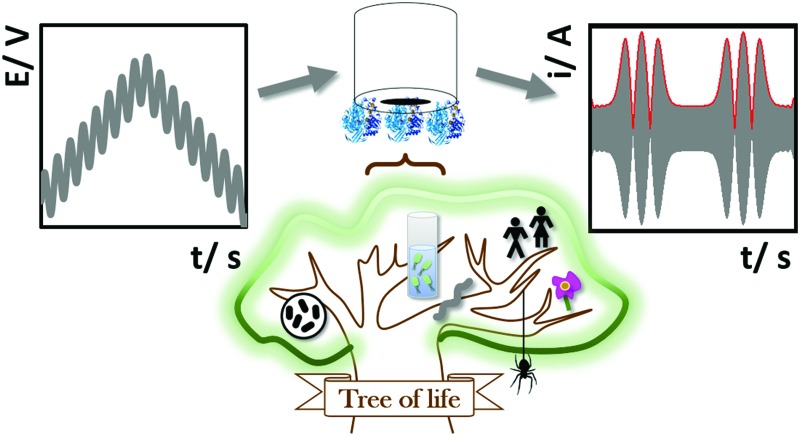
A review of the insight into biological redox chemistry which has been enabled by the development of large amplitude Fourier transform ac voltammetry.

## Introduction

**Fig. 1:**
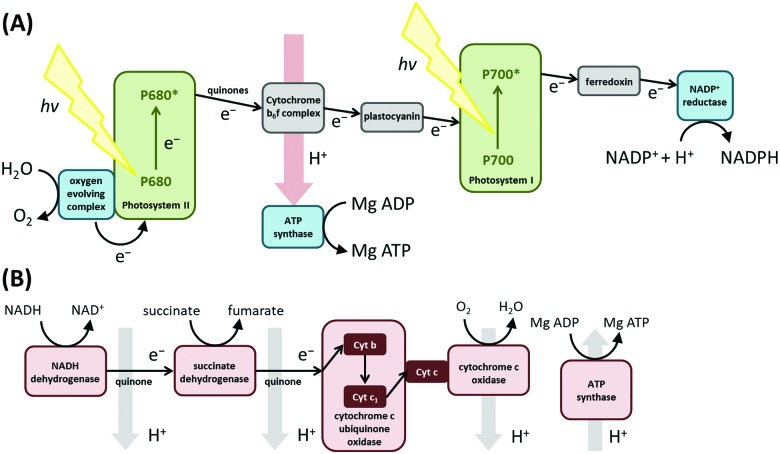
Biological electron transfer in (A) photosynthesis and (B) respiration.

Biological redox reactions are mediated by both small and large molecules, ranging from species such as quinones, which resemble typical synthetic chemistry redox reagents, to complex metalloenzymes comprising “wires” of redox-active centers (*i.e.* cytochrome *c* oxidase, the enzyme responsible for converting O_2_ to H_2_O in the mitochondria of all human cells, see Fig. 1).^1^ Redox reactions even underpin some DNA repair mechanisms.^2^,^3^ This review focuses on redox active proteins and enzymes, and the new mechanistic insights which can be gained by integrating the technique of large amplitude Fourier transformed voltammetry^4^ (FTacV) into the toolbox of protein film electrochemistry.

### Redox proteins and enzymes

The difference between a redox protein and a redox enzyme is that while the redox cofactors within the former simply act as electron transfer conduits, catalytic redox chemistry is observed to occur in the latter, *i.e.* the transfer of electrons to substrate(s) at an active site initiates a chemical reaction which yields product(s). Fig. 1 illustrates the vital role that redox proteins and enzymes play in mediating the biological energy transduction pathways of photosynthesis and respiration.^5^,^6^ While plastocyanin and ferredoxin are redox protein components of photosynthesis, photosystem II and NADP^+^ reductase are redox enzymes. (Photosystem I is a special class of light-activated redox protein.) Together, these electron transfer reactions provide most of the energy required for life on Earth. Mimicking such light-driven fuel production is a major challenge of modern scientific research, and understanding more about the fundamental design features of redox systems which have been carrying out this challenging chemistry for millennia is therefore of great interest. Specifically, the “Green Chemistry” credentials of most redox biocatalysis is to be greatly admired: redox active proteins and enzymes are built from sustainable elemental resources, and are optimized to function rapidly and efficiently at ambient temperatures and pressures, and using water as a solvent.

### Techniques to study redox proteins and enzymes

The redox-active molecular centers within a protein or enzyme can comprise amino acids, organic cofactors or transition metal active sites.^7^ Insight into the structural configuration of these centers can be provided *via* a number of techniques, with protein crystallography providing an invaluable “big picture” 3-D map of the whole structure that is particularly useful for relating evolutionary sequence changes to alterations in biochemical function. Modern molecular biology techniques have largely overcome the historic challenges inherent in producing enough pure protein/enzyme for crystallographic trials, and developments in cryo-EM are delivering another step change in structural techniques. However, such measurements only give static snapshots, rather than reactivity measurements.

Spectroscopic techniques such as UV/vis, FTIR or EPR can be advantageously “blind” to the bulk of the protein structure, instead reporting solely on electronic and structural transitions related to redox reactions in a protein or enzyme. A complication is that because of the inherent changes in electronic structure, most redox reactions involve a transition between a spectroscopically visible and a spectroscopically silent state. A further limitation of all these techniques is the difficulty in resolving dynamic information about redox reactivity, particularly when the source/sink of electrons to/from a protein or enzyme is a chemical reducing/oxidizing agent (experiments only provide insight into a narrow thermodynamic window of electromotive force, and rates are limited by diffusion). Conversely, the technique of protein film electrochemistry (PFE), where redox active proteins or enzymes are directly “wired” to an electrode surface, permits wide and precise control of the thermodynamic driving force of electron delivery/removal *via* control of the electrochemical potential of the electrode.^8^–^10^ A precise measure of electron flow rate is also yielded *via* the monitoring of the electrical current. PFE is therefore a valuable and well established tool within the portfolio of experimental methods used to interrogate redox active proteins and enzymes.^8^–^10^

This review aims to illustrate the limitations which arise from using conventional dc voltammetry in PFE, and exemplify the more powerful insight which can be gained from the development of protein film large amplitude Fourier transformed alternating current voltammetry (PF-FTacV).

## Protein film electrochemistry

### Protein immobilization

Historically, bio-electrochemistry proved challenging, as there were problems with protein denaturation at electrode surfaces and slow electron transfer.^8^ Small molecule electron transfer reagents (mediators) were used to shuttle electrons between the electrode surface and proteins or enzymes in solution.^11^ In such experiments, reactions are limited by the properties of the mediator and intrinsic protein/enzyme biochemistry cannot be measured directly. However, in 1977 Eddowes and Hill,^12^ and Yeh and Kuwana,^13^ separately demonstrated that reversible voltammetry of a solution of the heme protein cytochrome *c* is possible at electrodes of either bis(4-pyridyl) modified gold, or indium oxide, respectively. Further study showed that the reversible electrochemistry incorporated transient adsorption events at the electrode surface.^14^,^15^ This led to the strategy of directly immobilizing protein in an electroactive configuration at the surface of an electrode, and this technique has been popularized as “protein-film voltammetry”, then more generally “protein film electrochemistry” (PFE), by Armstrong and co-workers.^16^,^17^

**Fig. 2:**
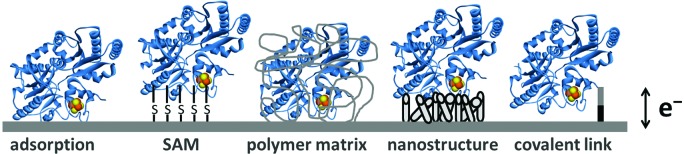
Protein–electrode attachment strategies.

Immobilizing a redox protein or enzyme removes the limitations of slow macromolecular diffusion which otherwise limits a solution experiment, and ensures the mechanism, thermodynamics and kinetics of electron transfer or redox catalysis intrinsic to the biological molecule can be exquisitely resolved using sub-picomole samples.^8^,^9^,^16^ The electroactive surface confinement of a redox active protein or enzyme, *i.e.* the attachment of the biomolecule to the electrode in a configuration that yields direct electron exchange, can be achieved *via* a number of methods (Fig. 2). The most common approach is the non-covalent adsorption of purified protein/enzyme onto an electrode, either an abraded pyrolytic graphite edge surface, a self-assembled monolayer on gold, a nanostructured material, or within a conducting polymer matrix (Fig. 2).^10^ Alternatively, covalent attachment linker chemistry is sometimes used.^18^ Aspects of the electrochemical response can be used to judge the efficacy of the surface confinement method (*vide infra*). A significant concern in PFE is that the fully assembled macromolecule structure is under interrogation, and not simply a protein-ejected/mis-folded redox active cofactor. Observation of electrocatalytic turnover allays such fears in the study of redox enzymes. The co-integration of the surface-mass measurement of quartz crystal microbalance with dissipation monitoring (QCM-D)^19^ or spectroscopic techniques^20^ such as surface enhanced infrared absorption (SEIRA)^21^ into redox protein and enzyme film electrochemistry studies has provided further evidence that proteins can be stabilized on electrodes in a fully competent configuration.

### Electrochemical cell setup

**Fig. 3:**
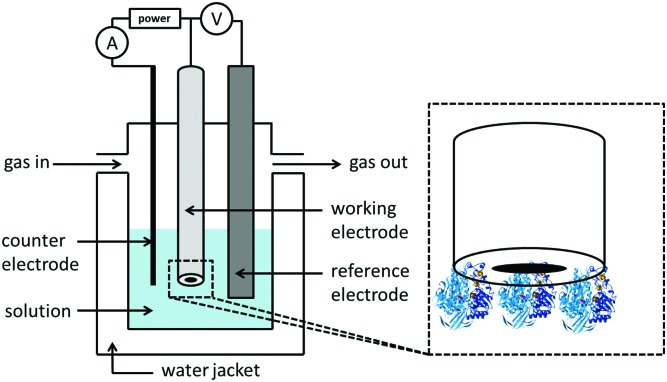
Protein film electrochemistry set-up.

Following the establishment of a film of redox protein/enzyme on a conducting surface, this “working electrode” is then placed in a solution filled electrochemical cell. The further addition of a reference and counter (also known as auxiliary) electrode yields the three-electrode set-up used in standard electrochemical measurements (Fig. 3). Potentials are applied between the working and reference electrodes and current flows through the counter electrode to minimize drops in potential due to solution resistance (ohmic drop) and prevent current flow through the reference electrode, which would alter its potential.^22^–^24^

The pH of the experiment is controlled *via* the composition of the electrochemical cell solution, into which soluble substrates or inhibitors can also be added at known concentrations.^22^ Most electrochemical cells are also designed to include a water-jacket, to facilitate control of the experimental temperature *via* use of a thermostated water heater/chiller re-circulation unit. The gas atmosphere can also be controlled *via* the addition of inlet and outlet gas lines to an otherwise sealed setup.^22^,^24^

## Protein film direct current cyclic voltammetry

**Fig. 4:**
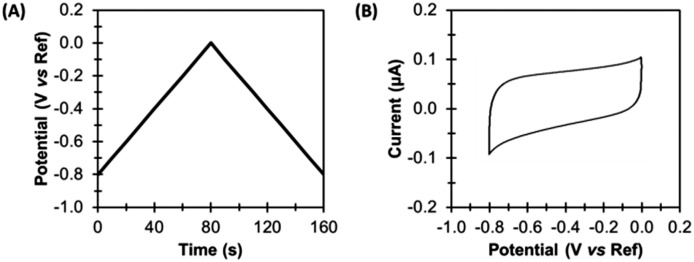
Direct current cyclic voltammetry (dcV). (A) Applied potential, and (B) non-Faradaic current.

In the simplest direct current cyclic voltammetry (dcV) experiment, the potential is either increased or decreased from a starting potential to a turning potential at a linear scan rate, before the direction of change is reversed, and the same rate of potential change is applied, but in the opposite direction.^23^,^25^ For example, Fig. 4 illustrates an experiment where the voltage applied to the working electrode at time zero (the starting potential) is –0.8 V *versus* the reference. A scan rate of 0.01 V s^–1^ is applied, so that after 80 s the turning potential of 0.0 V is achieved, at which point the scan direction changes. From 80–160 s the potential of the working electrode becomes more negative, until a voltage of –0.8 V is again attained. The resultant voltage–time waveform is often referred to as a saw tooth, and more complex waveshapes than that shown in Fig. 4A can be derived by introducing potential pre-poises and using different start and end potentials.^23^,^25^

A dcV cyclic voltammogram plot depicts the electrical current which passes between the working and counter electrodes in response to the applied potential, as shown in Fig. 4B. Electrical current can arise from both non-Faradaic and Faradaic (redox) processes.

### Non-Faradaic current

Non-Faradaic current arises due to the electrocapacitive charging and discharging of the double layer which forms at the electrode-solution interface in response to voltage changes.^23^,^25^ Such current is therefore an ever-present background contribution which is measured in all voltammetric experiments. Fig. 4 shows the capacitance-dominated current response of a typical pyrolytic graphite edge disk electrode of geometric surface area 0.03 cm^2^ when scanned with the dcV waveform illustrated.

### Faradaic current

Faradaic current arises from redox reactions at the electrode surface; with net reduction or oxidation giving rise to negative or positive currents, respectively.^23^,^25^ In protein or enzyme film dcV (PF-dcV), non-catalytic electron transfer reactions are conceptually the simplest redox reactions to study. However, in practical terms the measurement of Faradaic current arising from catalytic redox reactions is far simpler, and a large amount of the recent PF-dcV literature describes the analysis of such signals.^9^,^26^

### Non-catalytic current

**Fig. 5:**
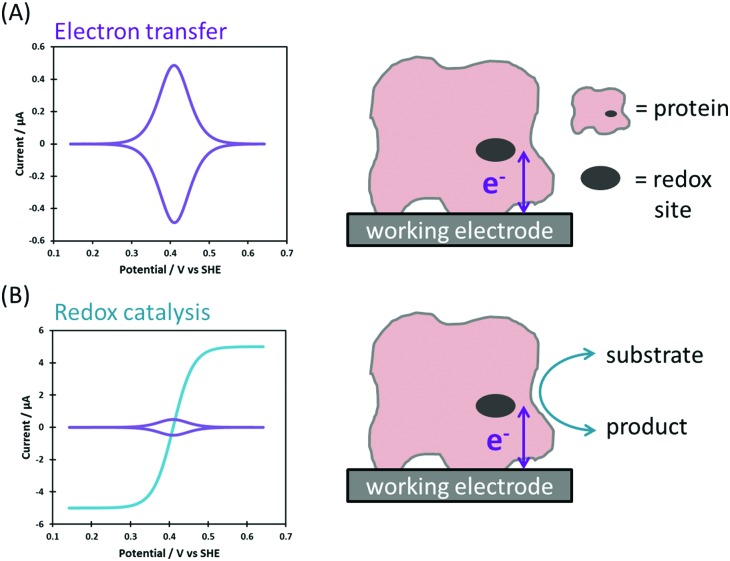
Ideal Faradaic-only current responses for (A) reversible electron transfer and (B) redox catalysis.

Non-catalytic current signals arise in PF-dcV as a result of the reversible reduction and oxidation of redox centers within a protein or enzyme.^9^ A single redox site with a reversible redox couple gives a pair of well-defined positive and negative current peaks in cyclic voltammetry, due to oxidation and reduction of the site, respectively (Fig. 5A).^9^,^16^,^27^ These so called “non-turnover” signals provide a thermodynamic description of the redox process. When the scan-rate is slow enough that the protein is under equilibrium conditions; the average peak potentials equal the reversible potential, the peak width at half-height measures electron stoichiometry and integration of the peak area quantifies the amount of electroactive protein adsorbed on the electrode.^9^,^16^,^27^ The situation is more complex in the case of proteins/enzymes with numerous redox-active sites, but there are numerous examples of elegant and insightful studies of such systems.^16^

Non-idealities in peak separation and width in slow scan rate dCVs are accounted for by considering that there will be a range of different protein orientations on the electrode, and this will give rise to dispersion in the thermodynamic and kinetic parameters.^28^,^29^

Faster dCV scan rates are used to probe the kinetics of the electron transfer reaction. The separation in the potentials of oxidative and reductive current peaks often allows the rate constant of intermolecular (electrode-to-protein) electron transfer to be approximated.^9^,^27^,^30^ If the reductive and oxidative peak currents are of differing magnitude, then the voltammetric response is reporting on the rate of coupled chemical steps that are slow on the time-scale of the experiment, for example proton-coupled electron transfer.^9^,^27^,^28^,^31^ Since the cell conditions can be rapidly titrated, *i.e.* a range of solution pH can be investigated quickly, the kinetics and thermodynamics, and thus mechanism, of electron transfer can be investigated using small samples of protein.

The limiting issue with such dc-CV experiments is that because the electrode surface coverage of proteins/enzymes is low, the Faradaic current from “non-turnover” redox reactions is small. In the best-case scenario, these small signals are superimposed on the vastly larger background capacitive current, and extensive baseline subtraction is therefore required, a process which introduces possible error into all subsequent analysis.^28^ In a significant number of cases the worst case scenario arises, and current arising from protein/enzyme reversible electron transfer processes is undetectable.

### Catalytic current

A redox enzyme adsorbed at an electrode will turn over substrate when the electrode potential provides sufficient driving force for catalysis (Fig. 5B). The net flow of electrons between the electrode and substrate results in the measured catalytic current, and this is proportional to the electroactive coverage of enzyme and the turnover rate.^9^ If the turnover rate is high, as is often the case for enzymes, the catalytic current is greatly enhanced relative to that arising from “non-turnover” processes, and this is referred to as a “catalytic amplification”.

To ensure that the turnover rate of electrocatalysis is limited by inherent enzymatic processes rather than experimental artefacts, the rate of interfacial electron transfer must be fast and mass transport of substrate must not be rate limiting. In the latter case, this is prevented by using a rotating disk electrode that is spun at a sufficient rate to ensure that the hydrodynamic flux of substrate no longer limits the current.^9^

In the simplest case, the catalytic current varies sigmoidally with electrochemical potential due to the Nernstian shift of the active site between its oxidized and reduced states (Fig. 5B).^32^ The catalytic current reaches a plateau, and this limiting value reflects the maximum enzyme turnover rate under the experimental conditions.^31^,^32^

The simple sigmoidal current response can be complicated by a number of factors,^9^,^26^,^33^,^34^ including intramolecular electron transfer (if there is a redox relay within the enzyme),^35^,^36^ substrate binding and release,^37^ (in)activation processes^38^,^39^ and dispersion of enzyme orientations on the electrode.^40^ The electrochemical response therefore reports on the thermodynamics and kinetics of many mechanistic aspects of electrocatalysis, which may be inaccessible by other techniques.^26^

Arguably, the most important insight into enzymology offered by catalytic voltammetry arises from the control of the potential domain. This not only enables activity to be surveyed as a function of redox state, it also permits quantification of the catalytic bias and overpotential requirement of the enzyme.^41^,^42^

**Fig. 6:**
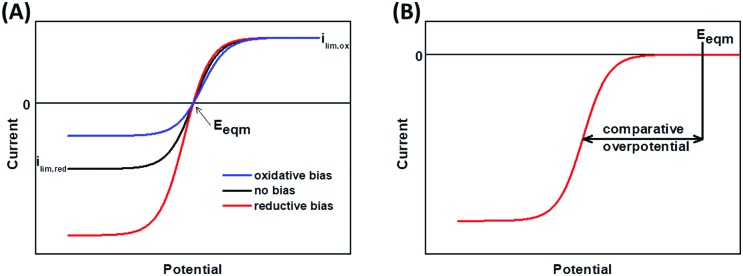
Voltammetric responses showing (A) catalytic bias and (B) overpotential.

Catalytic bias is defined as the ratio of the maximum limiting oxidation and reduction currents, and is therefore a measure of whether the enzyme is a faster catalyst of the reductive or oxidative reaction.^27^,^41^ Fig. 6A shows the voltammetric response from an enzyme biased towards oxidation (blue), reduction (red), and with no bias (black).

An overpotential requirement is defined as extra potential (or driving force) required by the enzyme to drive net oxidation or reduction of substrate, further to the potential defined by the Nernstian equilibrium potential of the substrate/product couple (Fig. 6B).^41^,^43^ It is often challenging to unambiguously determine the onset potential, *E*_onset_, of unidirectional Faradaic current because of the background non-Faradaic current, and double derivative analysis has been used to try and overcome this issue.^44^ Alternatively, representative “*E*_m_” catalytic potentials can be defined by measuring the potential of the maxima in a first derivative of the voltammogram, and this permits facile comparison of one enzyme to another. In the chemical redox catalysis literature, the potential at which a certain current density is achieved is often compared.

In PF-dcV, both the deconvolution of what underlying redox processes “gate” the catalytic reactions of an enzyme, and the conversion of catalytic current to turnover rates requires comparison of non-catalytic and catalytic voltammetry measurements. “Non-catalytic” conditions can be achieved in redox-enzyme measurements by removal of substrate, addition of inhibitor, or the use of high voltammetric scan rates that outpace catalysis but not electron transfer.^9^,^27^,^45^,^46^ However the limitations in obtaining clear non-catalytic signals, as outlined above, means such measurements often do not yield useable data, requiring the development of more sophisticated voltammetric measurement techniques.

## Alternative voltammetric waveshapes

There are numerous voltammetric techniques in which the voltage applied to the working electrode is periodically modulated as a function of time. This is done to enable at least partial separation of Faradaic and non-Faradaic current contributions within the resultant alternating current (ac) response. Such electrochemical techniques have also been used to enable discrimination of the current response from closely related mechanisms.^23^ Indeed the first two papers describing the electrochemical interrogation of reversible protein electron transfer utilized non-linear voltage sweeps.^12^,^13^

As noted by Elton and co-workers, there is no difference between different ac modulated-potential forms of voltammetry other than the exact form of the periodic perturbation to the linear potential sweep.^4^ However, traditionally the label “ac voltammetry” has been used to describe experiments that apply a small amplitude sinusoidal perturbation to the linear voltage sweep. The resultant ac component of the current is plotted against the mean (dc) potential of the sweep.^23^ Historically, such voltammetry has been used in mechanistic studies, as the dual time domain of dc scan and ac period provide greater access to precise kinetic and mechanistic information.^4^,^47^ However there is only limited differentiation between Faradaic and non-Faradaic (capacitive) current when small amplitudes are employed.^4^

The label “differential pulse voltammetry” (DPV) has been used to refer to experiments in which a series of large amplitude voltage pulses are superimposed on the linear potential sweep.^48^,^49^ Current is sampled immediately prior to each pulse and the current differences are plotted as a function of potential.^45^,^46^ Measuring current prior to the voltage steps minimizes the involvement of the capacitive charging current, which is generated by changes in potential.^45^,^46^ Alternatively, in “square-wave” voltammetry (SWV) a regular square-wave is superimposed onto a linear potential sweep, yielding a staircase voltage sweep. This can be considered as a special form of DPV in which equal time is spent at the ramped baseline potential and the pulse potential.^50^ Several papers have utilized square wave voltammetry to enhance protein electron transfer signals.^51^,^52^

## Fourier transformed ac voltammetry (FTacV)

### Development of PF-FTacV

The historical reason for the application of small amplitudes in sinusoidally-oscillating ac voltammetry was to simplify the analysis of results.^4^ Using such experimental parameters ensured that the resulting current trace could be accurately interpreted with analytical solutions because a small amplitude sinusoidally varying input potential only leads to a sinusoidal current response at the same frequency.^53^,^54^ In contrast, large amplitudes expose the non-linearity in the Faradaic *i*–*E* relationship, and the current response is no longer simply at the input frequency, *f*, but comprised of a series of sinusoidal signals at *n* × *f* of the input frequency, plus an aperiodic dc component near 0 Hz.^4^,^53^ The current component at *n* × *f* is referred to as the *n*th harmonic response. The limitation in being able to understand this current response was overcome in 2000 by Gavaghan and Bond^53^ and Engblom, Myland and Oldham,^54^ who contemporaneously presented generalised numerical and analytical solutions for large amplitude ac voltammetry. The large amplitudes classically used in DPV and SWV can therefore be utilized in all ac voltammetry.

Accessing the high harmonic signals that result from using large amplitude sine wave voltage-perturbations has a number of advantages, it enhances the experimentalists ability to accurately measure Faradaic current signals *via* the separation of capacitive and Faradaic current, and the separation of catalytic and electron transfer current, yielding concomitant improvements in mechanistic insight.^4^,^55^ The technique of large amplitude alternating current voltammetry has been advanced extensively by Bond and co-workers^4^,^55^,^56^ with the required instrumentation developed by Elton and co-workers.^4^,^56^,^57^ Modern computing allows fast Fourier transform methods to be used to separate components of the current output and therefore vastly improve the quality of data analysis and the kinetic, thermodynamic and mechanistic insight gained.^4^,^53^ This consolidation and development of large amplitude ac techniques is now collectively referred to as Fourier Transformed ac Voltammetry (FTacV).^4^ We use the phrase “PF-FTacV” to refer to the application of FTacV for the interrogation of protein or enzyme films. Although the advantages of FTacV have been well demonstrated for small redox molecules, the technique of PF-FTacV is still in its infancy.

### Experimental protocol

#### Applied potential

The 3-electrode electrochemical cell set-up of FTacV is the same as for dcV but a bespoke potentiostat is used to apply a waveform of frequency (*f*) and amplitude (Δ*E*) onto the dc voltage sweep of scan rate *ν*.^4^,^55^ A large amplitude sinusoidal waveform is most commonly used so that the applied potential, *E*_app_, is the sum of a dc component and an ac contribution as given by eqn (1) where *ω* = 2π*f* is the angular frequency of the sine wave.^4^,^58^,^59^
1
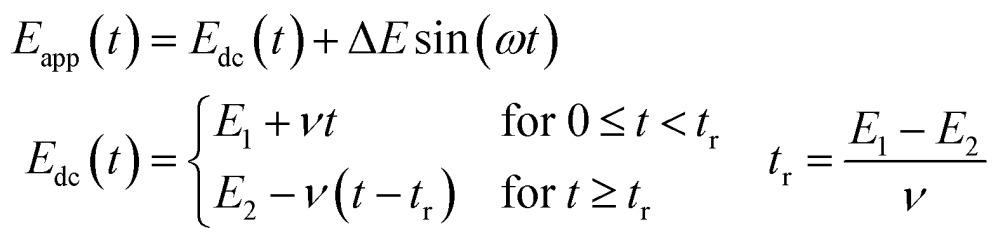



#### Fourier transform

**Fig. 7:**
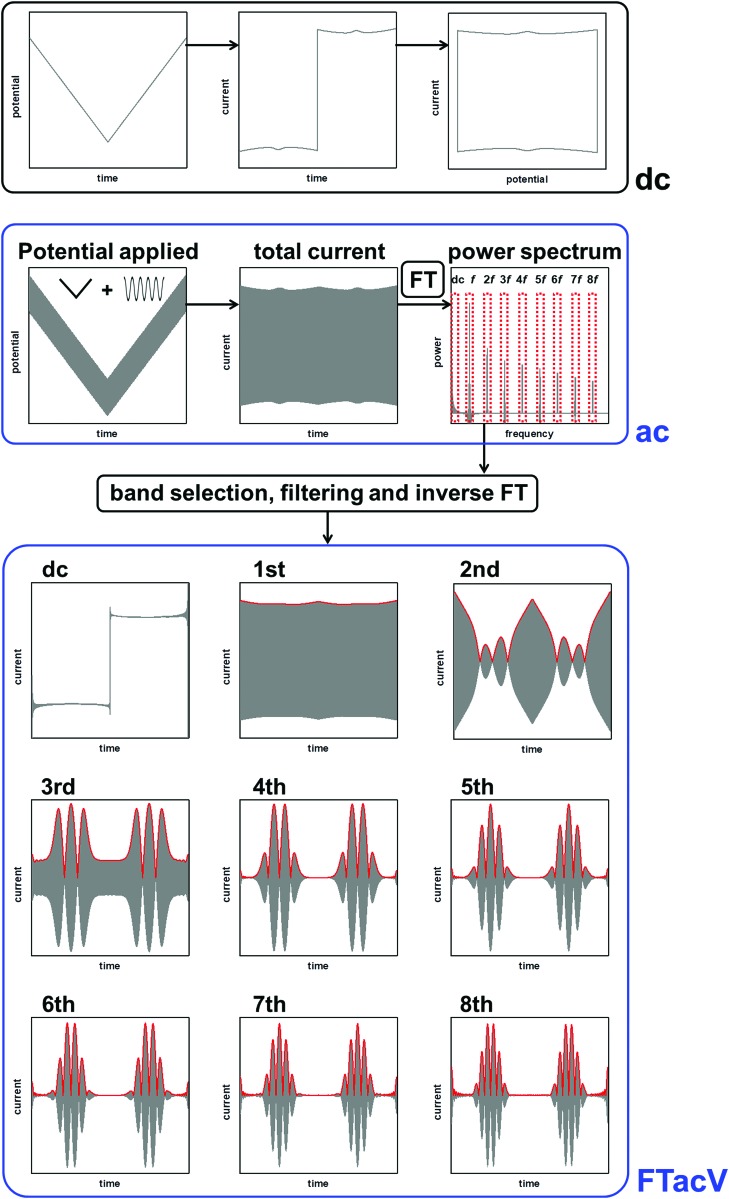
Comparison of FTacV and dc voltammetry techniques.

The total current output that results in response to the applied potential waveform is measured as a function of time and then Fourier transformed into the frequency domain to obtain a power spectrum (Fig. 7).^4^,^55^

A discrete Fourier transform (DFT) converts a finite set of equally spaced samples of a function into the frequency domain representation of the original input sequence. Eqn (2) describes the Fourier transformation of the sequence of N complex numbers *x*_0_, *x*_1_, …, *x*_*N*–1_.^60^,^61^
2
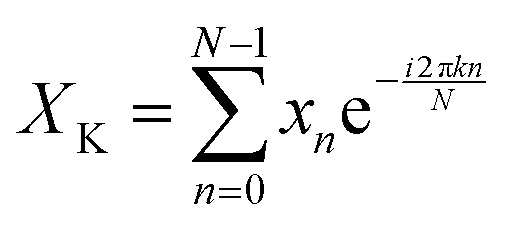
Direct evaluation of eqn (2) requires O(*N*^*2*^) operations, as there are *N* outputs and each output is a sum of *N* terms. Fast Fourier transforms use an algorithm to compute the same result in just O(*N *log* N*) operations.^62^ The Cooley–Tukey algorithm is commonly used and works by recursively re-expressing the DFT of a composite of size *N* = *N*_1_*N*_2_ as smaller DFTs of sizes *N*_1_ and *N*_2_, for *N* a power of 2.^63^ The FTacV potentiostat instrument automatically adjusts the dc scan rate and applied frequency so that 2^*n*^ data points are taken in a set. A fast Fourier transform (fFT) can then be performed *via* the Cooley–Tukey algorithm, to give the frequency domain representation.^56^

#### Band selection and inverse Fourier transform

From the frequency domain power spectrum that results from fFT, each individual component can be resolved by band selection (Fig. 7). The selected and filtered harmonic or dc component can then be resolved into the time domain by performing an inverse Fourier transform (iFT).^4^

A simple and commonly applied type of band selection involves the use of a rectangular window that leaves the frequency content within the window unchanged, and zero elsewhere. For example a rectangular function centered at *f* = 0 is defined in the frequency domain by eqn (3).^64^3
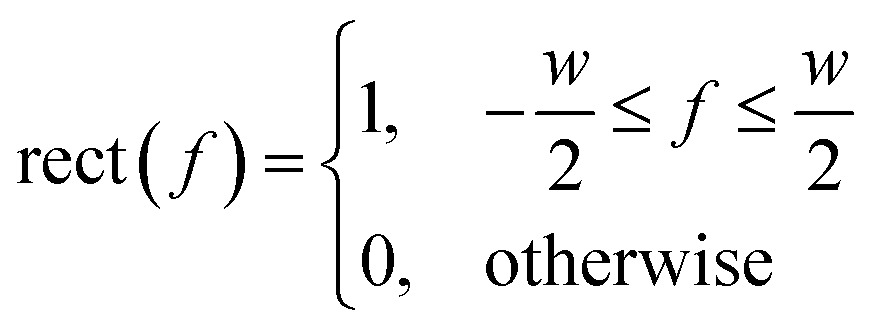
where *w* is the width of the window.

The iFT of this rectangular function is *R*(*t*) = *w *sin* c*(π*tw*) and the sin *c* function is defined by eqn (4).^64^ Such a window was used in the YedY FTacV experiments described later.4
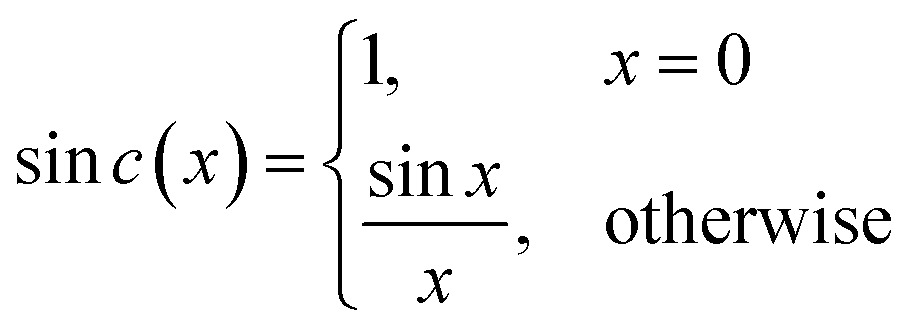
In some instances, using such a rectangular function can convolute the signal in the time domain, with the sin c function causing “ringing” artefacts which must be checked for by visual inspection.^64^ To avoid this, more sophisticated bell-shaped Kaiser or Hamming windows can be used, which remove the sharp discontinuities in the window function and reduce ringing artefacts.^64^ These more sophisticated windows were exemplified in the analysis of HypD and Hyd-1 described later.

#### Data plotting

Having carried out band selection and iFT, the current–time response of the aperiodic dc component and each harmonic signal is most commonly represented as either an absolute current magnitude envelope plot (red lines in bottom panel of Fig. 7), or the full current response is depicted (grey signals in bottom panel of Fig. 7).^4^,^55^ Analysis of the separate harmonic current components permits insight into both Faradaic and non-Faradaic processes and resolution of the catalytic and non-catalytic current from an enzyme.

The linear scan rate can be applied to convert time into average potential, and therefore plot a more classic current–potential *x*, *y* plot voltammetric response for each harmonic. In simulation studies, it is sometimes helpful to separate the current magnitude plots into the real and imaginary response.

## FTacV data analysis

### Non-Faradaic current

If the double layer capacitance at the electrode surface behaves as a simple capacitor then the capacitance current–potential relationship is given by eqn (5).5
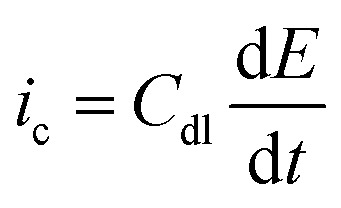
The capacitive current, *i*_c_, therefore has the response to the applied voltage-oscillation shown by eqn (6).^4^,^65^
6
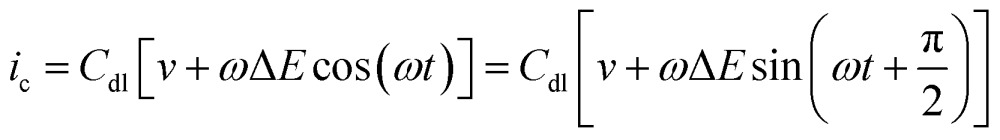
In this ideal scenario, the “background” non-Faradaic current response is at the same frequency as the sinusoidally varying input potential but phase shifted by π/2.^23^ The only harmonic ideal-capacitive response is therefore at the fundamental (1st) harmonic, and no higher harmonic current contributions are observed due to the linearity of the system.^4^

In reality, the double-layer capacitance at the graphite working electrode surfaces best suited to protein-film experiments is found to be potential dependent and the second, third and sometimes even fourth harmonics can therefore contain a capacitive component.^65^ There is no physical model for this potential dependent capacitance, and instead it is modelled with a polynomial, as shown by eqn (7).^65^7
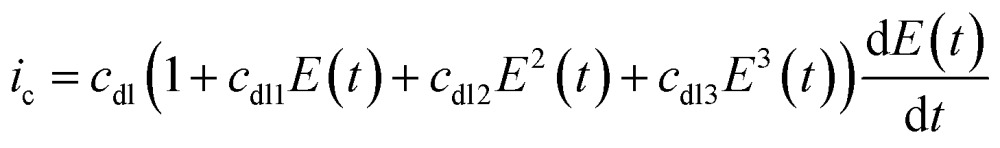



### Non-catalytic current

**Fig. 8:**
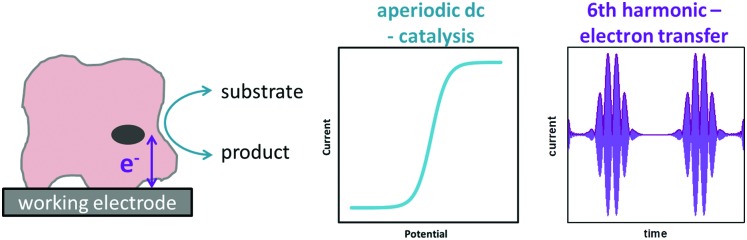
Deconvolution of catalytic and electron transfer current by FTacV.

As above, in general the current from fifth and higher harmonic signals is completely devoid of a capacitive component. Rapid, reversible redox processes have a non-linear response to the voltage oscillation, and so the resultant Faradaic current output has harmonic contributions at multiples of the input frequency (*f*, 2*f*, 3*f etc.*).^4^ Signals from the 5th harmonic upwards can therefore provide a baseline-free measurement of non-catalytic reversible electron-transfer redox processes in proteins and enzymes (Fig. 8).^55^ As described later, this improvement in signal clarity confers upon the experimentalist an enormous practical advantage of being able to detect Faradaic non-catalytic current even when the coverage of protein or enzyme on the electrode is so low that the signals would be invisible in dcV. The baseline-free intricacies of the high harmonic responses also enable clear discrimination between different mechanisms of electron transfer.^4^

The simplest case of simultaneous, non-catalytic transfer of *n*-electrons for a surface confined species interrogated by FTacV, ignoring capacitive current and Ohmic drop contributions, was first solved by Honeychurch and Bond,^58^ and eqn (8) describes the total Faradaic current output of the ac voltammetric experiment.8
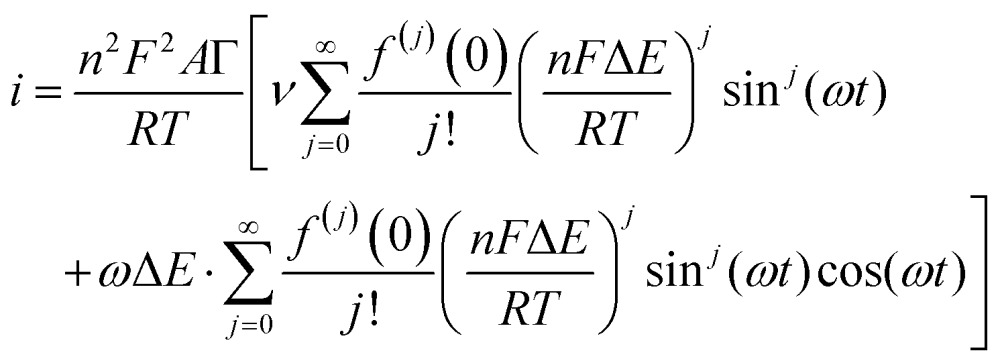
where *f*^(*j*)^ is the *j*th derivative of *f*(*a*).

Trigonometric identities are used to reduce sin^*j*^(*ωt*) and sin^*j*^(*ωt*)cos(*ωt*) to multiple harmonics and a dc term. When Δ*E* < 65 mV the dimensionless currents of the first three harmonics are described by eqn (9)–(11).^58^9
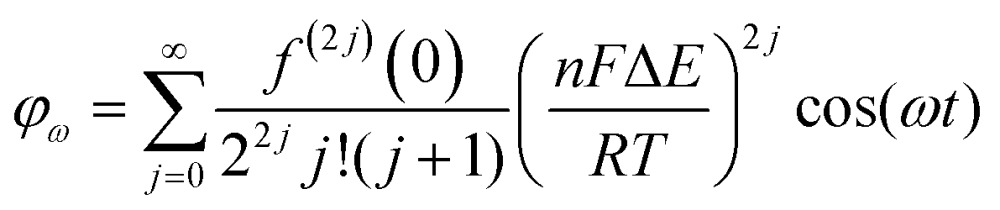

10
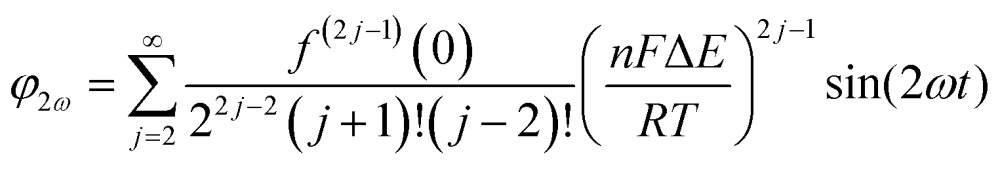

**Fig. 9:**
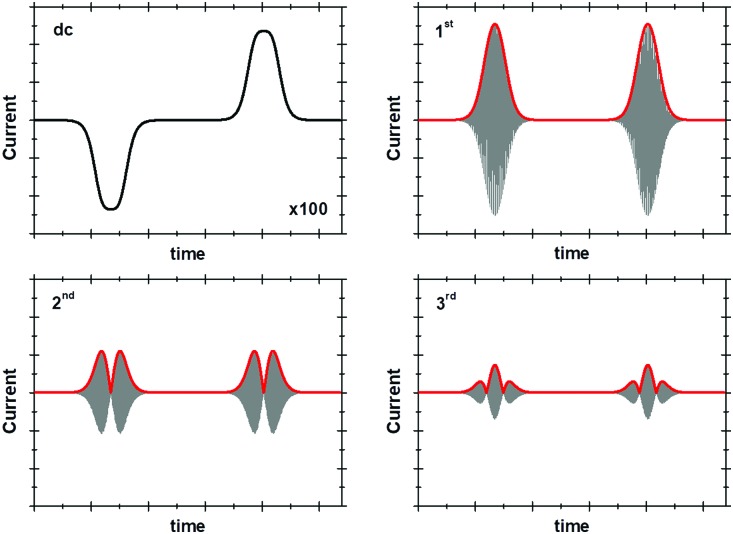
dc and 1st to 3rd harmonic components of a surface confined 1e^–^ transfer.

11
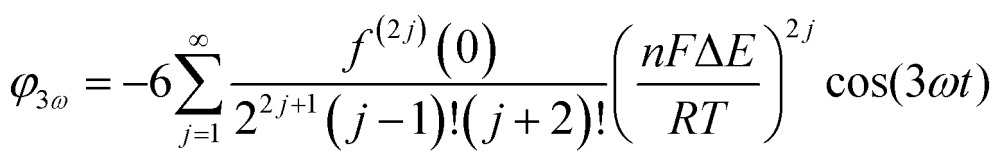
A representative example of the form that these ideal dc and harmonic Faradaic-only components take for a one-electron reversible electron transfer reaction is shown in Fig. 9. Whilst the main features of the aperiodic dc component are the same as expected from an equivalent dcV experiment some differences are observed since the ac perturbation causes peak broadening and, at larger amplitudes, peak splitting.^58^ The harmonic components, each centered at the reduction potential of the redox couple, decrease in current magnitude and split into further lobes at higher harmonics.^58^

The exact shape of each harmonic component is critically dependent on the mechanism and kinetics of the redox reaction and any coupled chemical processes.^55^ Cumbersome analytical solutions that are often only valid in certain limits are useful for visualizing and understanding the response but they are not used to analyze data. Instead, complete numerical simulations are used to model the data and determine the thermodynamic, kinetics and mechanism of the reaction.

### Catalytic current

An analytical solution presented by Zhang and Bond for the case of irreversible reduction catalysis by a surface confined species showed that the catalytic reaction does not affect the magnitude of the *n*th harmonic if *k*_red_ ≪ *nF*.^66^ In sufficiently high frequency experiments, the contribution of chemical catalysis to the high harmonics therefore becomes insignificant.^55^,^66^ In contrast, the aperiodic dc component preserves the main attributes and information available from a dcV experiment using the linear-only portion of the voltage perturbation. In enzymatic PF-FTacV, this portion of the data can therefore be analyzed to extract information on catalytic turnover, with experiments at different substrate or inhibitor concentrations giving similar insight into binding constants as can be derived from dcV. Some caution must be exercised, since broadening or further distortion may arise due to the large amplitude ac perturbation.^58^,^66^,^67^

Crucially, because a single enzymatic FTacV measurement provides a separate and simultaneous measurement of catalytic current (in the aperiodic dc-component), and baseline-free non-catalytic redox chemistry (in the high harmonic components), visual inspection of the data acquired in one experiment indicates the redox profile of the catalytic process and can quantify the redox potential(s) of any electron transfer step(s) (Fig. 8). This is hugely advantageous when compared to the substantial number of separate samples required to simply determine non-catalytic redox potentials *via* any spectroscopic redox titration. More detailed analysis of PF-FTacV permits even greater insight into the biological redox reactions, and numerical simulations are used to interrogate the thermodynamic, kinetic and mechanistic aspects of electron transfer reactions.^56^,^59^

## Numerical simulations of non-catalytic FTacV data

As described earlier, in PF-dcV the electron transfer kinetics of non-catalytic processes are probed by conducting multiple experiments at different scan rates, in order to compare the current response over a variety of different time-scales.^33^,^55^,^56^ By analogy, a single PF-FTacV experiment provides the same insight since each higher order harmonic essentially probes a shorter time-scale (higher frequency) current response than the one previously. Analysis of this data therefore permits the extraction of kinetic data free from the concern that film degradation has occurred over the course of running multiple separate dcV experiments.^55^,^56^

### Simulation methodology

In order to determine the thermodynamics, kinetics and mechanism of a reaction from voltammetric data, comparison to a numerical simulation is made.^26^,^59^ Robust commercially available simulation packages (such as DigiSim, DigiElch and KISSA) can simulate the dcV voltammetric response of a wide range of mechanisms.^59^,^68^ From the perspective of theory, ac voltammetry is closely related to dc voltammetry, and where dc simulations of an electrode process exist, an analogous computational procedure can be used to simulate ac voltammetry.^59^ The same equations describe the current output but the appropriate equation describing the applied potential in ac voltammetry is used. When analyzed *via* simulation the more information rich FTacV experiment can provide much greater discernment between different mechanisms and the effects of different parameters than dcV, ultimately providing a more accurate description of the electron transfer process.^56^

PF-FTacV presents a demanding situation for data simulation due to the small Faradaic signals and large background capacitive current, *i*_c_. The capacitive current cannot simply be ignored in simulations of high harmonics because it impacts on the total current (*i*), and therefore contributes to the ohmic (*iR*_U_) drop (*R*_U_ is uncompensated resistance). For example, for FTacV using a sine wave voltage oscillation the effective applied potential, *E*_eff_, is calculated using eqn (12) where *E*_dc_(*t*) is as described in eqn (1), *i*_c_ can be modelled by eqn (7), and Faradaic current, *i*_f_, is given by the appropriate equation for the redox reaction studied.^59^ The associated electron transfer rates in the Faradaic current equations can be described by either Butler–Volmer or Marcus–Hush–Chidsey formalisms.^59^

12 *E*_eff_(*t*) = *E*_dc_(*t*) + Δ*E *sin(*ωt*) – (*i*_c_ + *i*_f_)*R*_u_

To increase the efficiency of the numerical simulation, the problems are recast as dimensionless equations using dimensionless variables.^53^,^58^ The equations are solved by discretizing time-derivatives, so that the dimensionless current at each step *i* + 1 can be calculated using values from the previous step *i*.^53^,^58^ The simulation model and input parameters (*i.e.* capacitive, kinetic, thermodynamic values *etc.*) are varied until a satisfactory agreement is established between the experimental data and the simulated data.^4^,^56^ In PF-FTacV, since the aperiodic dc and lower harmonic components are more sensitive to the capacitive current, and the higher harmonics more sensitive to the Faradaic current, a two-step approach is used in this parameter optimization stage. Initially, the capacitive parameters are estimated from a fit to data present in the fundamental harmonic component over potential regions where its contribution is dominant relative to the Faradaic current (Faradaic current ideally absent). Subsequently the Faradaic parameters are derived on the basis of an optimised fit of experimental and simulated higher order harmonic components that are devoid of and significant capacitance background current.

### MECSim

MECSim is a freely available software package that enables most aspects of ac voltammetry to be simulated, including incorporation of uncompensated resistance, non-linear background capacitance and a choice of Butler–Volmer or Marcus–Hush–Chidsey electron transfer kinetics.^59^ The program supports numerical simulation of not just the ac voltammetric response of simple, reversible surface confined redox reactions (*i.e.* as described by eqn (8)),^58^ but also solution phase reactions,^53^ multi-electron processes,^69^,^70^ chemically coupled reactions^59^ and many other mechanisms.^59^ The kinetic schemes can also be modified to factor in dispersion.^29^ MECSim was used to perform the YedY simulations described later. The simulation parameters were optimized to give a best fit to the experimental data *via* a heuristic approach, *i.e.* each individual parameter was manually adjusted.

### Automated parameter optimization

Heuristic parameter optimization to yield the best fit between simulated and experimental data is a time-consuming and potentially biased process.^56^,^71^ An alternative approach is the development of automated multi-parameter data optimization procedures that can be used to find the combination of parameters that formally minimize an objective function (a mathematical representation of the quality of the fit).^72^–^74^ This automated approach towards finding the combination of parameters which yield the minimal difference between simulated and experimental data has been used to simulate HypD and Hyd-1 PF-FTacV data, as described later.^75^–^77^

### Using simulations of non-catalytic current to determine catalytic turnover rates

Although the focus of PF-FTacV simulations to date has been the modelling of non-catalytic redox reactions, this process is still useful in the analysis of catalytic data because one of the “fit” parameters is the molar amount of electroactive protein/enzyme on the electrode, *M*. Therefore, in the case of enzymatic PF-FTacV experiments when a non-catalytic, reversible redox reaction is observed in the high harmonics and catalytic current is measured in the aperiodic dc-component, the limiting catalytic current “plateau” value (*i*_lim_) can be converted to a corresponding limiting enzymatic turnover rate (*k*_lim_) once the high harmonic signals have been accurately simulated to yield *M*.^51^,^62^ This conversion is done using eqn (13) where *F* is the Faraday constant and *n*_cat_ represents the number of electrons transferred in the catalytic reaction.

13 *i*_lim_ = *k*_lim_*Mn*_cat_*F*

The direct measurement of a turnover rate in enzymatic PF-FTacV represents a significant advantage over enzymatic PF-dcV experiments. Using PF-dcV, it is only possible to determine the coverage of enzyme on the electrode *via* non-catalytic measurements. As detailed above, the ratio of Faradaic to non-Faradaic (capacitive) current in such experiments is often too low to yield meaningful data. Even if the coverage can be determined from such a dcV experiment, a separate measurement must then be conducted to measure the catalytic current, introducing unknown inaccuracies to the measured turnover rate.^9^ Additionally, the possible impact of substrate binding on non-catalytic electron transfer rates and kinetics cannot be tested in PF-dcV, but can be determined using PF-FTacV.

## Primary phase PF-FTacV studies: technique development

The first stage of any technique development is the performance of proof-of-concept calibration experiments which quantify the accuracy and ability of the new methodology. The first tranche of PF-FTacV experiments therefore represent this challenging, exciting and enriching primary phase of research and development, and prove that FTacV can be utilized as a powerful and experimentally efficient tool for probing biological redox chemistry.

### Azurin: an excellent model protein for probing one-electron reversible biological redox reactions

Because the aim of the first, pioneering PF-FTacV experiments was to probe a redox active protein that has well characterised redox chemistry, the chosen subject was the blue copper protein azurin.^78^ This structurally-simple, mono-copper containing electron-transfer protein has been extensively investigated with dcV and represents something of a model system for biological one-electron transfer, with the copper center capable of rapidly transitioning between the +1 and +2 oxidation states.^16^,^30^,^78^–^80^

The first azurin PF-FTacV experiments proved that in sine-wave measurements the Faradaic current from reversible electron transfer can be isolated from the background non-Faradaic/capacitive current.^78^ The effects of scan rate, amplitude and frequency were also explored, and previously developed theoretical models^58^ were successfully applied to simulate the experimental data.^78^

Further studies on azurin were undertaken using PF-FTacV in its square wave form.^81^,^82^ In such experiments the even harmonics could be shown to contain all the current arising from quasireversible processes, while the odd harmonics measure current arising purely from background and reversible processes, providing exquisite kinetic evaluation.^81^,^82^

Returning to sine wave azurin PF-FTacV, studies then showed that the higher harmonic signals (fourth and above) provide the best separation of background and Faradaic current, and the differing kinetic sensitivity of each harmonic can be used to measure electron transfer rates.^83^ Simulations showed non-idealities in the experimental data which were attributed to a dispersion in the orientation of enzyme molecules on the electrode surface.^83^ In a separate study, that used PF-FTacV to compare the response from azurin adsorbed onto different length alkanethiols, it was shown that it is possible to discriminate between enzyme-surface orientations with different interfacial electron transfer rates.^84^

### Heme containing proteins and enzymes

Having used azurin as an excellent model system with which to explore the utility of PF-FTacV for probing non-catalytic biological redox chemistry, cytochrome P450s were then chosen as sample heme-containing biological redox systems which have been extensively studied with other electrochemical techniques. Heme-centers are multi-functional iron-sites which are ubiquitous across life, playing essential enzymatic and O_2_-storage roles that fundamentally depend on the control and tuning of the redox chemistry of the metal site.

In the first PF-FTacV study of a cytochrome P450 it was experimentally proven that irreversible O_2_ reduction catalysis and the underlying heme reversible electron transfer can be separated into the aperiodic dc and higher harmonic components, respectively, of a single measurement.^85^ This observation has since been rationalized *via* theory.^66^ This powerful ability to interrogate catalytic and non-catalytic redox processes simultaneously remains one of the most significant advantages of PF-FTacV over PF-dcV and redox-titration spectroscopy methods.

In the second cytochrome P450 PF-FTacV study, the technique was showcased as a tool to directly measure the heme midpoint potential *via* simple inspection of the background-free 4th harmonic component, *i.e.* unlike PF-dcV, complex baseline subtraction was not required.^86^ The most recent 2015 PF-FTacV paper on a cytochrome P450 utilized the kinetic sensitivity of the method to show that electron transfer rates may be modified by protein–protein interactions.^87^

Continuing with heme protein redox analysis, PF-FTacV has also been used to compare myoglobin and free heme, showcasing the information-rich higher harmonics as a sensitive measure of experimental subtleties.^88^ A PF-FTacV study of the heme enzyme cytochrome *c* peroxidase provided data against which a theoretical mechanism for sequential two one-electron transfer to/from a single surface confined center could be tested.^69^ The higher harmonic components were found to have enhanced sensitivity to the nuances of such two-electron transfers, and heuristic simulations were used to determine the level of cooperativity in the individual one-electron steps, as well as the kinetics and thermodynamics.^69^ Again, dispersion was deemed to be responsible for non-idealities in the experimental data.^69^

### PF-FTacV interrogation of multi-electron transfers

The limitation of azurin and single-heme proteins/enzymes as PF-FTacV test systems is that they do not require the development of more highly complex simulation models. Further PF-FTacV technique development studies have therefore focused on the interrogation of more complex biological redox systems which have previously been amenable to detailed PF-dcV measurements and therefore have well understood redox chemistry.

A ferredoxin was chosen as an example of an electron-transfer protein containing two separate iron–sulfur redox-active centers which both have very similar redox potentials.^70^ This study demonstrated that PF-FTacV can be used to resolve Faradaic signals from two such centers, and the theory needed to simulate the experimental response from such surface-confined two-center metalloproteins was also developed.^70^ Heuristic simulations were used to calculate the kinetics and thermodynamics of electron transfer to/from each iron sulfur cluster.^70^

The two-electron redox chemistry of the organic FAD center of glucose oxidase, an enzyme most beloved in the electrochemical sensor community ever since it was incorporated into diabetic glucose blood monitors by Hill and co-workers, was also studied using PF-FTacV.^8^,^75^ The higher harmonics were again used to effectively discriminate between different possible mechanisms of two-electron transfers. It was shown that the FAD undergoes two consecutive one-electron transfers at approximately the same potential, rather than one simultaneous two-electron transfer.^75^ This study also introduced the use of Marcus theory in simulations, and the use of automated E-science approaches, rather than heuristic simulation-parameter optimization, to find the best combinations of up to three variables.^75^

## Secondary phase PF-FTacV studies: acquiring otherwise unobtainable insight into biological redox chemistry

Since 2015, a secondary phase of PF-FTacV research has been initiated *via* collaboration between the Bond group in Monash University and the Parkin group at the University of York.^89^–^91^ Following our earlier work,^69^,^70^ we (Parkin and Bond) are now seeking to showcase FTacV as the first choice electrochemical technique for the interrogation of new biological redox chemistry, utilizing this technique for studying important redox proteins/enzymes and seeking to develop mechanistic insight which is otherwise unobtainable from other electrochemical techniques. Instead of using well established, existing models of redox biochemistry to calibrate the PF-FTacV technique, this new phase of work is using PF-FTacV as the frontline, go-to measurement to ask questions to which the answer is otherwise unknown. A crucial aspect of this flourishing area of work has been the further improvement in simulation methodologies provided through a very valuable collaboration with the Gavaghan group in Oxford.^90^,^91^ The remainder of this paper will present the major findings from Bond and Parkin's collaboration to date in the form of three “case studies” which each act as a brief precis of the individual studies.

## Case study 1, PF-FTacV applied to enzymology: deciphering the catalytic mechanism of *Escherichia coli* YedY

**Fig. 10:**
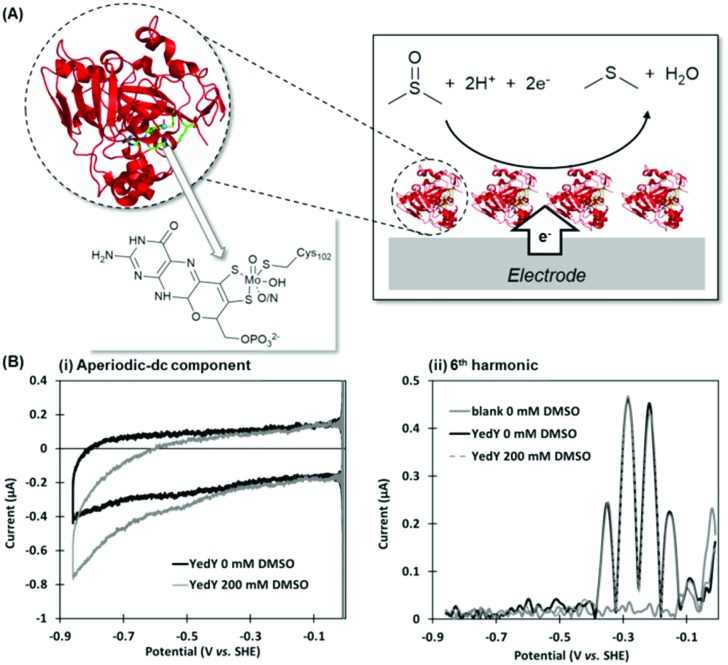
(A) Crystal structure of *Escherichia coli* YedY (PDB ; 1XDQ) including active site detail and cartoon depiction of DMSO electrocatalytic reduction reaction. (B) Simultaneous measurement of catalytic and non-catalytic redox chemistry *via* PF-FTacV.

*Escherichia coli* YedY is a mononuclear Mo-enzyme which is an exemplar of a bio-catalyst produced by many different bacteria (Fig. 10). Recent work has shown that the reason so many bacteria produce one of these types of enzyme is because it catalyzes the reduction of methionine sulfoxide, *i.e.* this enzyme is produced in response to oxidative stress to repair bacterial protein structures.^92^

**Fig. 11:**
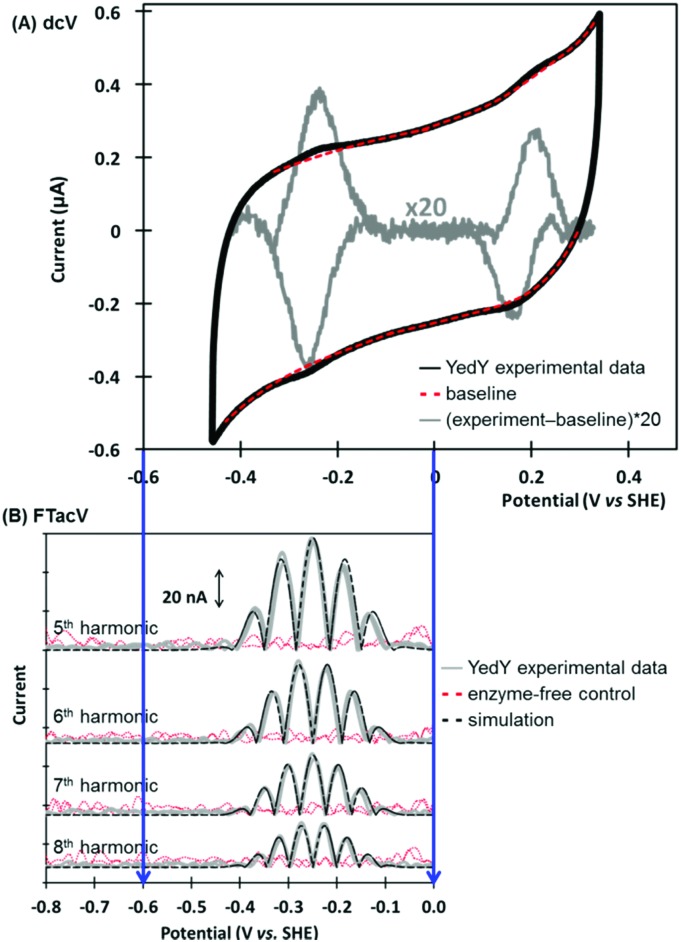
Non-catalytic redox activity of *Escherichia coli* YedY as assessed by (A) dcV, and (B) PF-FTacV.

PF-FTacV was utilized to fully characterize the kinetics, thermodynamics and mechanism of a previously unknown two-electron two-proton redox reaction of YedY.^89^ This redox chemistry could be clearly distinguished from the higher potential, one-electron Mo^5+/4+^ redox transition which was also visible in electrochemical measurements and had previously been characterised using EPR (Fig. 11).^89^ Since the protein structure only possesses one cofactor (Fig. 10), the two-electron redox reaction was therefore attributed to the organic pyranopterin ligand which binds to the Mo. Such “non-innocent” ligand-based redox chemistry has been predicted by small molecule studies of molybdopterin complexes, but had not been previously observed in biochemical measurements. Crucially, the ability of FTacV to simultaneously measure catalysis and electron transfer in a single experiment showed that the proposed pyranopterin redox chemistry “gates” the catalysis of the substrate mimics DMSO and TMAO, *i.e.* the two-electron active site reduction must occur before substrate activation can commence (Fig. 10). This PF-FTacV study therefore provides the first electrochemical evidence that pyranopterin redox activity can be catalytically relevant to the enzymatic activity of a Mo enzyme, rather than just *in vitro* small molecule mimic studies.^89^ This settles an ambiguity in how YedY, which displays only one-electron Mo chemistry, can catalyse two-electron reactions usually performed by Mo^4+/6+^ transitions in other molybdoenzymes.

The MECsim FTacV data simulations performed in this study played a vital role in concluding that the low potential process arose from the rapid transfer of two electrons *via* two sequential one-electron steps, enabling comparison between one-electron and simultaneous two-electron transfer mechanisms. The utility of the simulation package was therefore highlighted. Practically, conducting this study also highlighted the time-consuming nature of heuristically optimizing the simulation parameters, emphasizing the urgent need for computational modelling in our next study.

## Case study 2, data optimization of PF-FTacV simulation parameters: interrogating disulfide bond redox chemistry in *Escherichia coli* HypD

**Fig. 12:**
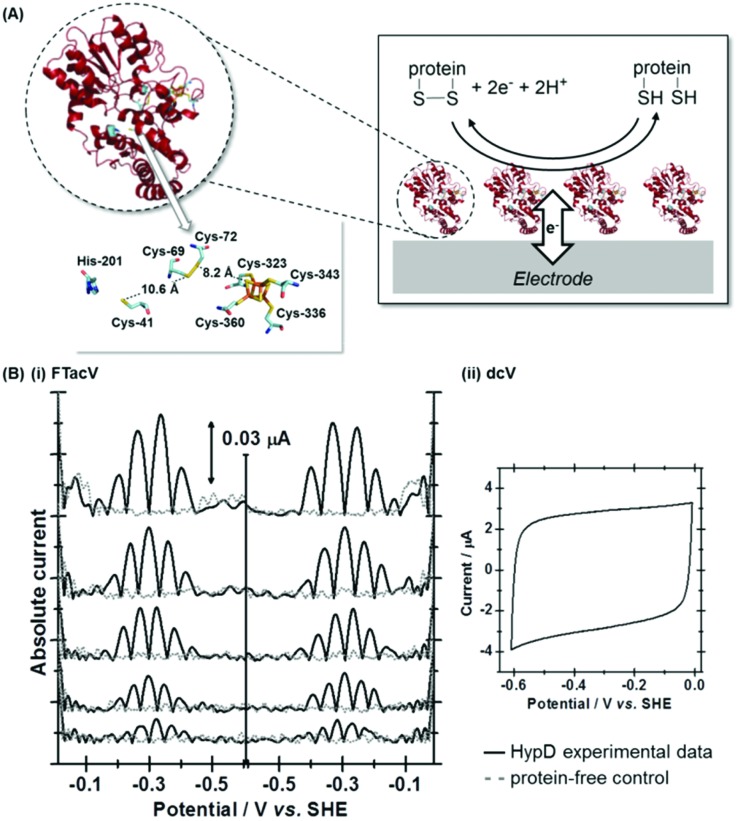
(A) Structure of *Escherichia coli* HypD including detail of postulated reaction centers, and cartoon depiction of reversible disulfide redox reaction. (B) Comparison of the measurement sensitivity of (i) high harmonic PF-FTacV signals *versus* (ii) dcV.

A study of *E. coli* HypD used PF-FTacV to quantify the thermodynamic, kinetic and mechanistic aspects of a previously uncharacterized cysteine disulfide redox reaction.^90^ The protein also contains a [Fe_4_S_4_] cluster which previous spectroscopic studies had designated as redox inactive. HypD is part of the biosynthetic machinery that builds [NiFe]-hydrogenases in *E. coli*, and the electrochemical study aids understanding of how cyanide ligands may be reductively inserted into the hydrogenase active site. The PF-FTacV work showed, for the first time, that the signal enhancement and background current discrimination possible with FTacV is such that redox signals can be observed from a protein with PF-FTacV even when no discernible signal can be measured with standard PF-dcV (Fig. 12).

**Fig. 13:**
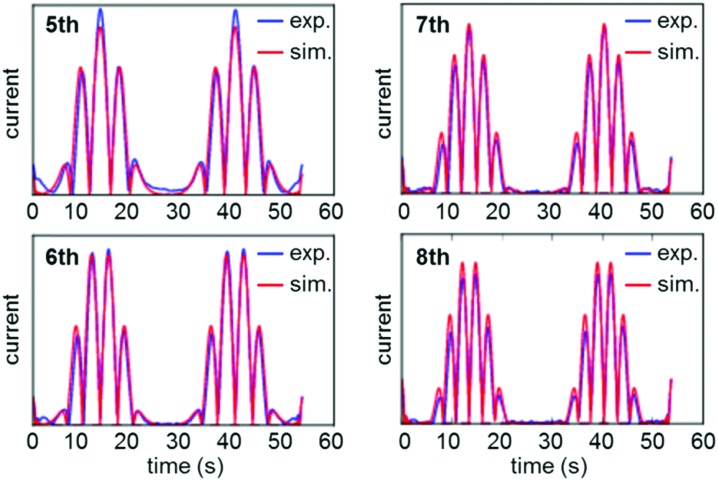
Overlay of experimental (exp.) and data-optimized simulation (sim.) of high harmonic FTacV data for *Escherichia coli* HypD.

Rather than relying on the type of heuristic simulations used to fit the YedY PF-FTacV data, the HypD study describes a two-step automated parameter optimization methodology.^90^ This computerized procedure for FTacV simulations has been specifically developed to deal with the large-capacitance issues which relate to using graphite electrodes for protein adsorption.^90^ The simulation methodology enabled the efficient comparison of the HypD experimental data to the best fit obtained from three possible model redox reaction mechanisms, either a one-electron transfer, one simultaneous two-electron transfer, or two separate, sequential one-electron transfers. The good fit between the experimental data and the latter-most model (Fig. 13) supports the conclusion suggested by inhibitor experiments, that the cysteine-bridge is the only redox active structural motif in the form of the protein which was being interrogated. An obvious direction for future work is to therefore probe whether metal binding of partially constructed hydrogenase active site activates electron transfer to/from the iron–sulfur cluster.

## Case study 3, separating enzyme wiring from fast, reversible proton catalysis: *Escherichia coli* Hyd-1

**Fig. 14:**
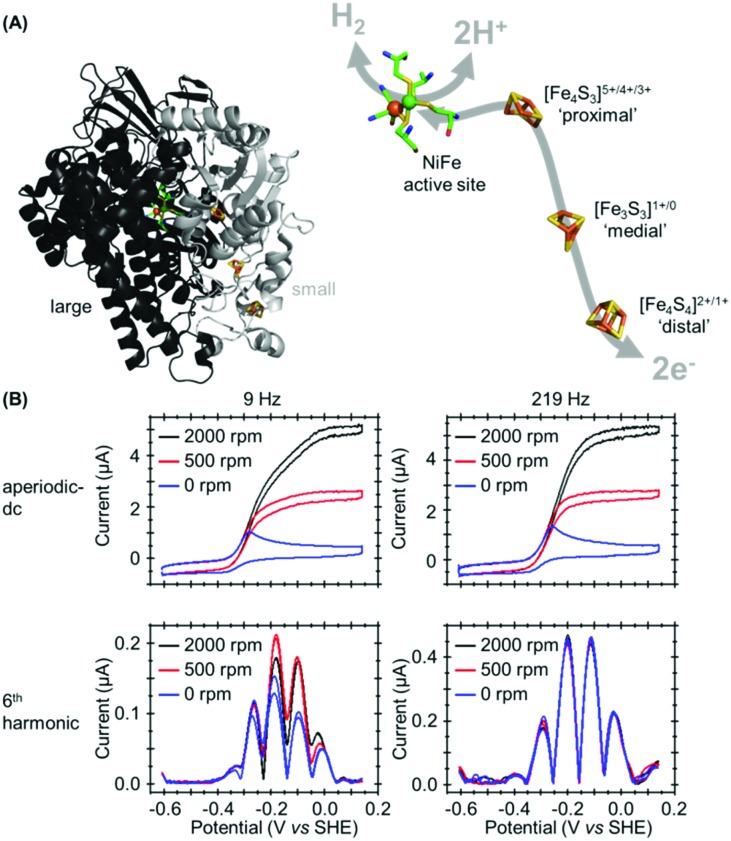
(A) Crystal structure of *Escherichia coli* Hyd-1 (PDB ; 3UQY) including detail of the redox active metal centers. (B) High frequency is required to separately isolate the catalytic and non-catalytic redox reactions.

In our most recent study we have utilized PF-FTacV to separate the catalytic and electron transfer components of the Faradaic current from a [NiFe]-hydrogenase, an enzyme capable of reversible H_2_ redox catalysis (H_2_ ⇌ 2H^+^ + 2e^–^).^91^ Using PF-dcV such a feat is impossible because protons cannot be excluded from water and therefore aqueous solvents in a biologically relevant pH range always contain substrate. With hydrogenase FTacV, we find that a sufficiently high frequency must be applied so that the high harmonic signals contain purely non-catalytic current. As shown in Fig. 14, at 9 Hz the 6th harmonic current remains sensitive to electrode rotation rate, indicating substrate turnover is detected. This ceases to be an issue at 219 Hz, when catalysis is outpaced by the higher frequency.

**Fig. 15:**
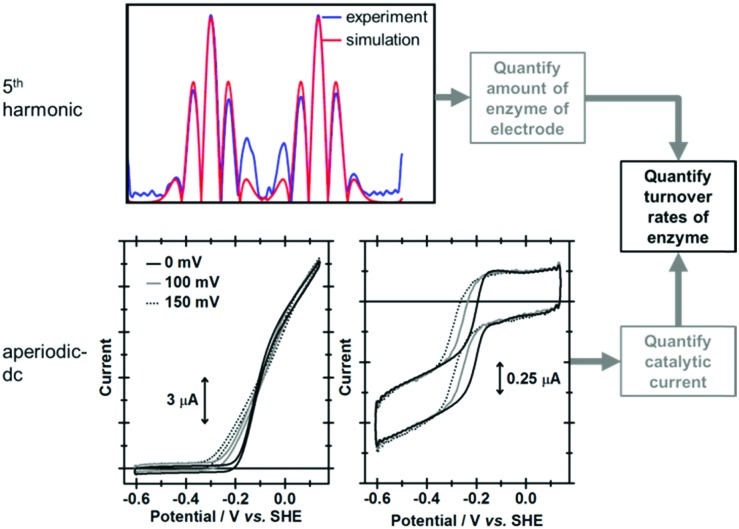
Illustration of how simulation of the high harmonic FTacV signals permits extraction of enzyme turnover rates from simulation of the aperiodic-dc catalytic currents.

True discrimination between catalytic current and non-catalytic, reversible electron transfer is confirmed by comparing the PF-FTacV of the native enzyme to the signals obtained from with an inactive enzyme variant.^91^ Simulations assist in the assignment of the non-catalytic electron transfer reaction as arising from the [Fe_4_S_4_]^2+/1+^ redox transition of the outermost “distal” cluster, and provide a method for quantifying the amount of protein on the electrode surface (Fig. 15). Again, the automated parameter optimization protocol is underpinned by a two-step procedure, in which capacitive parameters are fit using the lower harmonics and Faradaic parameters fit using the higher harmonics. As a further method development, thermodynamic dispersion is also incorporated into the model.^91^ The potential of the distal electron entry/exit site is re-tuned *via* an Arg to Leu amino acid exchange in the second coordination sphere of the iron–sulfur center. The simultaneous measure of catalytic current (in the aperiodic dc component) and enzyme coverage (from the high harmonic electron transfer signals) afforded by PF-FTacV enables the absolute turnover rate of the native and “R193L” amino acid variant enzyme to be calculated (Fig. 15), an impossibility in dcV experiments. *Via* such PF-FTacV we therefore directly relate the distal cluster redox potential change to the re-tuning of catalytic bias, and prove that the mutations have led to the production of an enzyme that is more biased towards H_2_ production.^91^

The aperiodic-dc data in Fig. 15 also illustrates the impact of sine-wave amplitude on the apparent onset of catalysis. For this reason, PF-dcV experiments were also conducted to determine the impact of the amino acid exchanges on the catalytic overpotential. It was therefore shown that compared to native Hyd-1, the R193L variant has a reduced overpotential for H_2_ oxidation at pH > 5. We could therefore prove we have artificially created a more thermodynamically optimized H_2_ oxidation bio-catalyst.^91^

## Conclusions

Although the original, seminal protein electrochemistry work of Eddowes and Hill^12^ and Yeh and Kuwana^13^ utilized alternating current techniques, it seems it is the perceived complexity of implementing ac measurements and interpreting the resultant data that has prevented the widespread use of ac methods in protein electrochemistry. Instead, PF-dcV measurements have dominated the field of PFE, and such studies have blazed a trail, establishing film-electrochemistry as a powerful and important technique for interrogating redox active biomolecules. We have now shown that as a further improvement, PF-FTacV greatly improves the clarity of protein electron transfer signals and enables enhanced kinetic and mechanistic insight into enzymatic and non-catalytic redox biochemistry. There remains an essential need to interpret such electrochemical results in light of complementary spectroscopic and structural data, and molecular biology is an essential tool which can assist in assigning reactivity to specific reaction centers. However, with PF-FTacV it is now possible to “see” otherwise invisible redox processes, and gain simultaneous catalytic and non-catalytic mechanistic insight in a single experiment. The technique of PF-FTacV is therefore a new and powerful addition to the complementary toolkit of techniques which can be used to understand the electronic circuitry of life.

It must be acknowledged that not all redox proteins can be adsorbed to electrodes in an electroactive configuration, so many are not amenable to electrochemical study. A key area of future work is therefore the development of more robust protein–electrode “wiring” approaches. The simulation of PF-FTacV data also remains an exciting and potentially very rewarding aspect of method development. Instrument development also remains an ongoing area of interest as ever more challenging biological systems require access to a greater number of harmonics and a wider range of amplitudes and frequencies.

Looking forward, we ultimately hope to see PF-FTacV enter a “tertiary phase” where any redox active protein or enzyme can be interrogated on any electrode surface *via* a user-friendly software interface. Ideally, the simulation parameters of a wide range of reaction models will be automatically adjusted to provide a facile comparison between experimental data and the “best fit” outcomes of numerous possible reaction mechanisms. Ideally, the integration of statistical methods should provide a way to quantify the accuracy with which each “best fit” parameter is known. In this endeavor, we are greatly assisted by the continual, rapid improvement in electronic components and computational hardware, the collaborative ethos with which engineers and computational scientists will share their expertise, and the growing appreciation which experimental chemistry researchers have for the essential utility of modelling tools.

Full knowledge of the electron transfer and catalytic properties of redox proteins and enzymes is key to understanding crucial life processes, and for learning how to design future energy bio-inspired or bio-based technologies. It is hoped that by continuing to advance the experimental and analytical methods of PF-FTacV, we will continue to learn more about the redox reactions of life.
